# Cascade degradation of organic matters in brewery wastewater using a continuous stirred microbial electrochemical reactor and analysis of microbial communities

**DOI:** 10.1038/srep27023

**Published:** 2016-06-07

**Authors:** Haiman Wang, Youpeng Qu, Da Li, John J. Ambuchi, Weihua He, Xiangtong Zhou, Jia Liu, Yujie Feng

**Affiliations:** 1 State Key Laboratory of Urban Water Resource and Environment, Harbin Institute of Technology. No. 73 Huanghe Road, Nangang District, Harbin 150090, China; 2 School of Life Science and Technology, Harbin Institute of Technology. No. 2 Yikuang Street, Nangang District, Harbin 150080, China

## Abstract

A continuous stirred microbial electrochemical reactor (CSMER), comprising of a complete mixing zone (CMZ) and microbial electrochemical zone (MEZ), was used for brewery wastewater treatment. The system realized 75.4 ± 5.7% of TCOD and 64.9 ± 4.9% of TSS when fed with brewery wastewater concomitantly achieving an average maximum power density of 304 ± 31 m W m^−2^. Cascade utilization of organic matters made the CSMER remove a wider range of substrates compared with a continuous stirred tank reactor (CSTR), in which process 79.1 ± 5.6% of soluble protein and 86.6 ± 2.2% of soluble carbohydrates were degraded by anaerobic digestion in the CMZ and short-chain volatile fatty acids were further decomposed and generated current in the MEZ. Co-existence of fermentative bacteria (*Clostridium* and *Bacteroides*, 19.7% and 5.0%), acetogenic bacteria (*Syntrophobacter*, 20.8%), methanogenic archaea (*Methanosaeta* and *Methanobacterium*, 40.3% and 38.4%) and exoelectrogens (*Geobacter*, 12.4%) as well as a clear spatial distribution and syntrophic interaction among them contributed to the cascade degradation process in CSMER. The CSMER shows great promise for practical wastewater treatment application due to high pre-hydrolysis and acidification rate, high energy recovery and low capital cost.

Large quantities of waste effluent are produced from brewing, cooling and washing units in beer brewing process, therefore, the treatment and safe disposal of brewery wastewater has become an important aspect because of the strict discharge regulations^1^. Brewery wastewater are easily biodegradable (BOD_5_/COD > 0.5) and nontoxic containing high content of carbohydrate and protein, which would be suitable for biological treatment^2^. Conventional treatment technologies such as aerobic sequencing batch reactor (SBR) and upflow anaerobic sludge blanket (UASB) have proven successful in treating brewery wastewater on industrial scale in the past decades^3^. However, energy input and sludge handling from aerobic treatment process significantly raise the projected cost of system operation^4^. In addition, requirements for thermophilic temperatures and low energy output efficiency for anaerobic treatment are still barriers that hinder its application on wider scope^5^. Therefore, it is paramount to develop energy- efficient treatment method for brewery wastewater.

From the perspective of energy-efficiency and energy recovery, microbial electrochemical systems (MESs) provide a solution for environmental sustainability by extracting energy from organic materials present in wastewaters while simultaneously removing pollutants^6^. A large number of real wastewaters have been explored as feed. Food industry wastewater, including dairy wastewater^7^, starch processing wastewater^8^, chocolate industry wastewater^9^, potato processing wastewater and olive mill wastewater^10^, have proved to be suitable for electricity generation in MESs due to their food-derived nature and lack of high concentrations of inhibitor substances. Among these food industry wastewaters, brewery wastewater has been intensively studied both in our lab and other research groups. The first paper using brewery wastewater as feed in a single-chambered air-cathode MES was published in 2008^11^, operation parameters including temperature and conductivity were optimized. Also, a 90-liter stackable pilot MES achieved net electrical energy harvest of 0.034 kWh m^−3^ when treating raw brewery wastewater^12^. Evaluation of long-term performance conducted in a 10-liter serpentine-type MES demonstrated that deterioration in cathode was the main reason for the decrease in performance over time^13^. These previous studies focused on electrochemical performance and final substrate removal efficiency, however, degradation process of macromolecular organic compounds in brewery wastewater and the associated functional microbial community distribution were not fully discussed.

Adding pre-hydrolysis and fermentation processes in MESs creates an opportunity for enhanced removal of COD when treating high strength wastewater comprising of complicated constituents^14^. Syntrophic interactions between non electrochemically active microorganisms and exoelectrogens are critical for such MES-centered systems, especially when they deal with complex substrates^15^. Due to possible multiple syntrophic processes occurring in fermentable substrate-fed MES-centered systems, including syntrophic interactions between fermenters and exoelectrogens, fermenters and methanogens, homoacetogenic bacteria and exoelectrogens, a wider range of substrates can be utilized by MES-centered systems than single MES^16^.

An integrated continuous stirred microbial electrochemical reactor (CSMER) developed by integrating CSTR and MES achieved a COD removal of 87.1% with energy recovery of 32.1% when treating sucrose wastewater at an organic loading rate of 12 kg COD m^−3^ d^−1^^17^. Although the CSMER successfully treated synthetic wastewater, further research is necessary to address the performance of treating complex soluble and suspended organics in practical wastewater, which might promote the practical application of MES-centered systems.

To investigate the performance of CSMER for complex organic compounds treatment, real brewery wastewater was used as feed in this study. It brought to view a better understanding of cascade utilization of macromolecular organic compounds in brewery wastewater and analyzed spatial distribution of microbes to clarify relationship between microbial community and substrate degradation mechanism. In addition, the CSMER was compared with a control reactor (continuous stirred tank reactor, CSTR) operated parallelized in terms of substrate removal and microbial community. Finally, it conducted comprehensive comparison of CSMER and other MES-centered systems and traditional anaerobic treatment process evaluating the potential for practical application.

## Results and Discussion

### Electricity generation from brewery wastewater

The CSMER operated at a hydraulic retention time (HRT) of 12 h with external resistance fixed at 10 Ω during the three experiment phases. Since the system had already run for 120 days with synthetic wastewater, current output of each cell reached to 17.8 ± 1.5, 17.6 ± 1.4, 16.4 ± 1.9 and 18.1 ± 1.6 mA immediately after it was fed with the influent containing 30% (V/V, in volume) brewery wastewater in Phase I. As the brewery wastewater concentration gradually increased from 30% to 100%, a reduction of 31.2% in mean current output was observed, with each cell stabilized at 12.8 ± 2.0, 11.4 ± 1.5, 10.6 ± 1.4 and 13.2 ± 1.2 m A in Phase III (Table S1). The reduction in current might be caused by more total suspended solid (TSS) contained in the influent, which increased from 451 ± 78 mg L^−1^ in Phase I to 1546 ± 136 mg L^−1^ in Phase III. Various colloidal particulates in the real wastewater would have negative effects on electricity generation as they are the main rate-limiting and resistance-increasing factors^18^.

**Figure 1:**
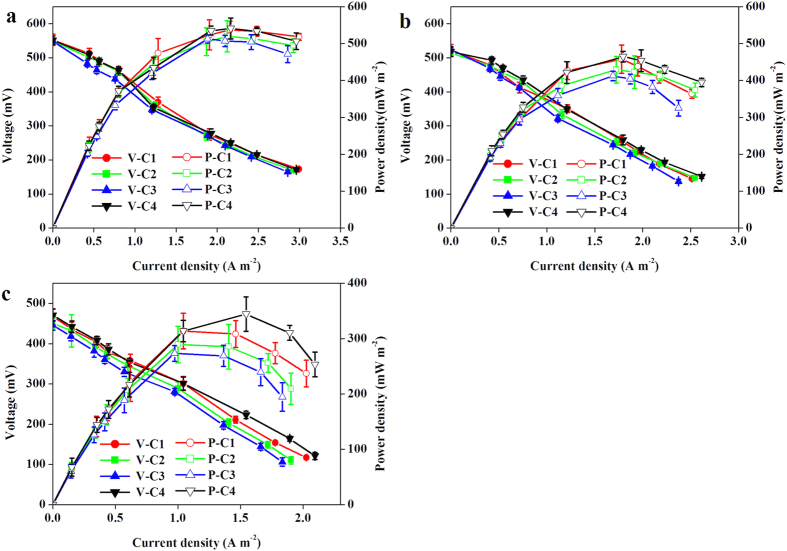
Power density and polarization curves of the CSMER in (**a**) Phase I (**b**) Phase II and (**c**) Phase III (V: voltage, P: power density, C1: Cell 1, C2: Cell 2, C3: Cell 3, C4: Cell 4).

Based on polarization data, the maximum power densities for each cell of the CSMER were 536 ± 9, 519 ± 18, 512 ± 23 and 541 ± 22 mW m^−2^ in Phase I (Fig. 1a). They also exhibited a decline trend as the brewery wastewater concentration increased, which were 313 ± 32, 289 ± 34, 273 ± 14, 344 ± 31 mW m^−2^ in Phase III (Fig. 1c). The slight differences in power generation of the four cells in the same phase were mainly caused by cathode according to the polarization curves, while insignificant change of anode potential was expressed (Figure S1). Nonuniform biofilm formation or salt precipitation on the cathode might lead to different cathode performance^19^. The mean maximum power density achieved by CSMER was 1.5 times higher of a single-chambered, air-cathode MES (205 m W m^−2^) fed with brewery wastewater^11^. This could be due to the syntrophic processes that occurred in the CSMER, in which better-utilizable substrates for electricity generation were provided through pre-hydrolyzing and acidifying complex organic substrates in brewery wastewater.

Coulombic efficiency (CE) was 1.5 ± 0.5% based on TCOD consumed in the whole CSMER, which calculated to be 4.3 ± 0.9% based on TCOD consumed in the microbial electrochemical zone (MEZ). This value was generally in agreement with other MESs tests using real wastewater^20^. The low CE could be related to interference of fermentation, methanogenesis and other biological processes in microbial electrochemical zone (MEZ), which were non-electricity production processes and eventually decreased the CE^21^. Suppression of methanogens without affecting exoelectrogens is of great importance for improving CE. Operating the CSMER under a lower external resistance or in open-closed circuit regimes could be a way to avoid CE losses to methanogenesis^22^. In addition, reduction of oxygen diffusion by employing a separator cathode assembly configuration could prevent aerobic oxidation of COD to some extent^23^. Furthermore, shortening the HRT of MEZ by enlarging the volume ratio of CMZ (complete mixing zone) and MEZ was another way for higher CE^18^.

**Figure 2:**
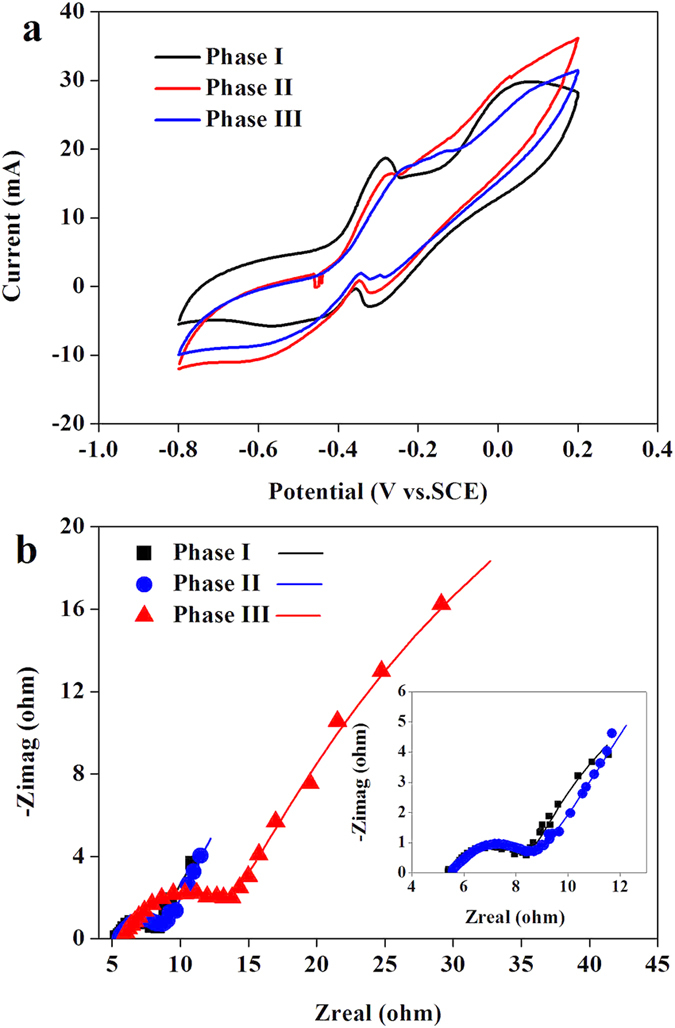
(**a**) Cyclic voltammogram of anode biofilm (Cell 1) and (**b**) Nyquist plots of electrochemical impedance spectroscopy of cathode (Cell 1) during the three operation period.

Cyclic voltammetry (CV) was performed on anode biofilm to evaluate its bioelectrocatalytic activity in the three phases. Since anode performance of the four cells was basically the same (Figure S1), CV analysis was only applied to one cell (Cell 1) of the system. In Phase I, significantly high oxidation and reduction peaks were observed in the forward scan of −0.28 V (18.7 mA) and reverse scan of −0.32 V (−2.9 mA) indicating the highest electrochemical activities of anode biofilm in this phase (Fig. 2a). The peak current slightly declined to 16.1 mA and 0.9 mA as the brewery wastewater concentration increased from 30% to 60%, which might be caused by a reduction of electrochemically active bacteria cell density on the anode surface due to more refractory organics present in the feed^24^. As the brewery wastewater concentration further increased to 100%, the peak current was similar as that in Phase II, indicating a functionally stable biofilm capable of adapting to complex substrates in brewery wastewater had developed on the anode surface. Pre-hydrolyzing and fermenting complex organic matter to short-chain fatty acids through the CMZ might reduce the inhibition effects of refractory organics on anode biofilm.

Electrochemical impedance spectroscopy (EIS) was conducted to determine the resistance of cathode reaction. The Nyquist plots of Cell 1 obtained in each phase indicated that total internal resistance started to increase in Phase II (Variations of the other three cathodes also followed the same trend). The ohmic resistance (*R*_ohm_) was not significantly changed due to the same system configuration, which was about 5.7 ± 0.3 Ω (Fig. 2b). However, the charge transfer resistance (*R*_c_) and the diffusion resistance (*R*_d_) increased from 2.7 ± 0.9 to 7.8 ± 1.2 Ω and 3.2 ± 0.6 to 17.6 ± 2.1 Ω as the brewery wastewater concentration increased from 30% to 100%. The increase in *R*_c_ and *R*_d_ might be a result of cathode biofouling, which was caused by biofilm formation or salt precipitation on the catalyst layer of cathode and consequently increased proton transport resistance or decreased oxygen diffusion rate^19^. Employing a separator electrode assembly configuration might be an efficient way to slow down the deterioration process of cathode performance^23^.

### Organic matter degradation and cascade utilization

#### Removal of COD and SS

Effluent total COD (TCOD) and soluble COD (SCOD) of the CSMER were monitored during the three - phase operation period. TCOD removal slightly decreased from 83.2 ± 1.3% to 79.1 ± 3.4% and SCOD removal decreased from 80.0 ± 3.1% to 76.8 ± 3.5% while the percentage of raw brewery wastewater in the feed was increased from 30% to 60%. The system removed 75.4 ± 5.7% of TCOD and 73.1 ± 4.8% of SCOD in Phase III when raw brewery wastewater was used, with a reduction of the TCOD concentration from 3707 ± 220 to 909 ± 296 mg L^−1^ and the SCOD concentration from 2466 ± 189 to 663 ± 117 mg L^−1^ (Table S1). Both TCOD and SCOD removal decreased as concentration of raw brewery wastewater kept increasing in the feed, indicating that the performance of CSMER got suppressed due to the induced substrate inhibition^25^. However, treatment efficiency of CSMER was still significantly higher than that of the control CSTR, which removed 47.2 ± 6.9% of TCOD and 38.4 ± 5.1% of SCOD in Phase III, resulting in final concentration of 1946 ± 186 and 1503 ± 230 mg L^−1^ in the effluent.

**Figure 3:**
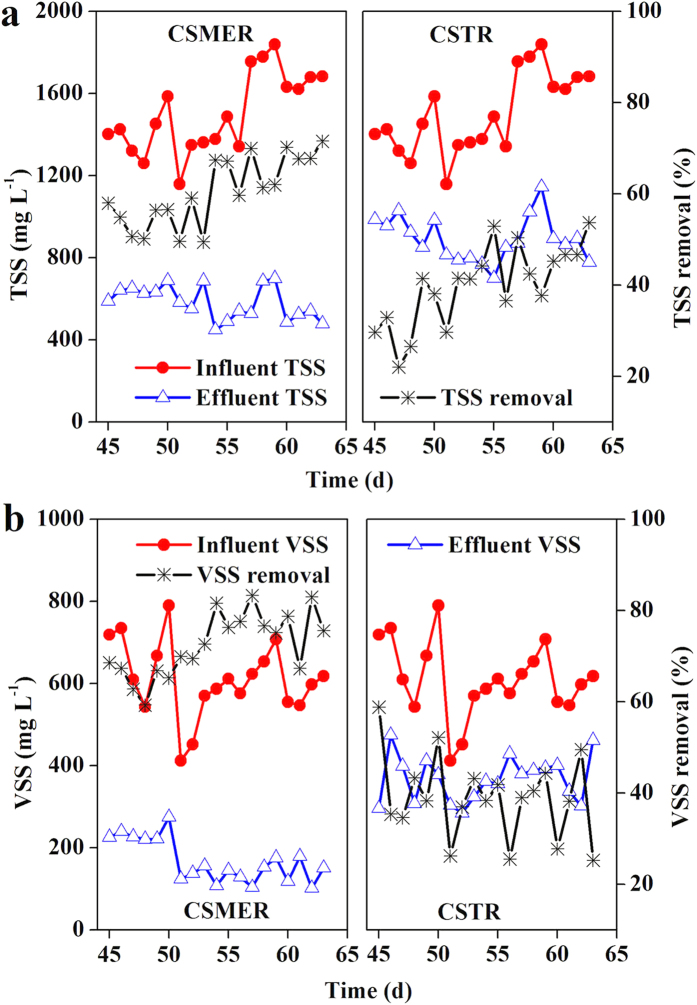
Variation of (**a**) TSS (**b**) VSS concentration in the influent and effluent of CSMER and CSTR in Phase III.

Suspended solid is an important indicator in biological wastewater treatment. The CSMER reduced 64.9 ± 4.9% of TSS and 72.8 ± 5.2% of VSS in Phase III, resulting in the effluent concentration of 538 ± 64 and 156 ± 50 mg L^−1^. Comparatively, the SS concentration in CSTR effluent was 862 ± 79 mg L^−1^ of TSS and 347 ± 61 mg L^−1^ of VSS (Fig. 3). In addition, higher methane production rate was obtained in the CSMER (0.41 ± 0.05 L L^−1^ d^−1^) than that in CSTR (0.25 ± 0.07 L L^−1^ d^−1^). Though there might be a competition between methanogens and exoelectrogens for the same substrates, interactions between methane production in the CMZ and electricity generation in the MEZ had a positive effect on substrate removal. Continuous methane production could accelerate the acetogenic process, which produced more hydrogen or VFAs. A portion of the VFAs could transfer to MEZ along the hydraulic path, where they were further utilized by exoelectrogens for electricity production. VFAs removal from the CMZ released their inhibition on methanogens, consequently, promoting the organic matter eventual decomposition to CO_2_ and H_2_O.

#### Removal of proteins, carbohydrates and VFAs

**Figure 4:**
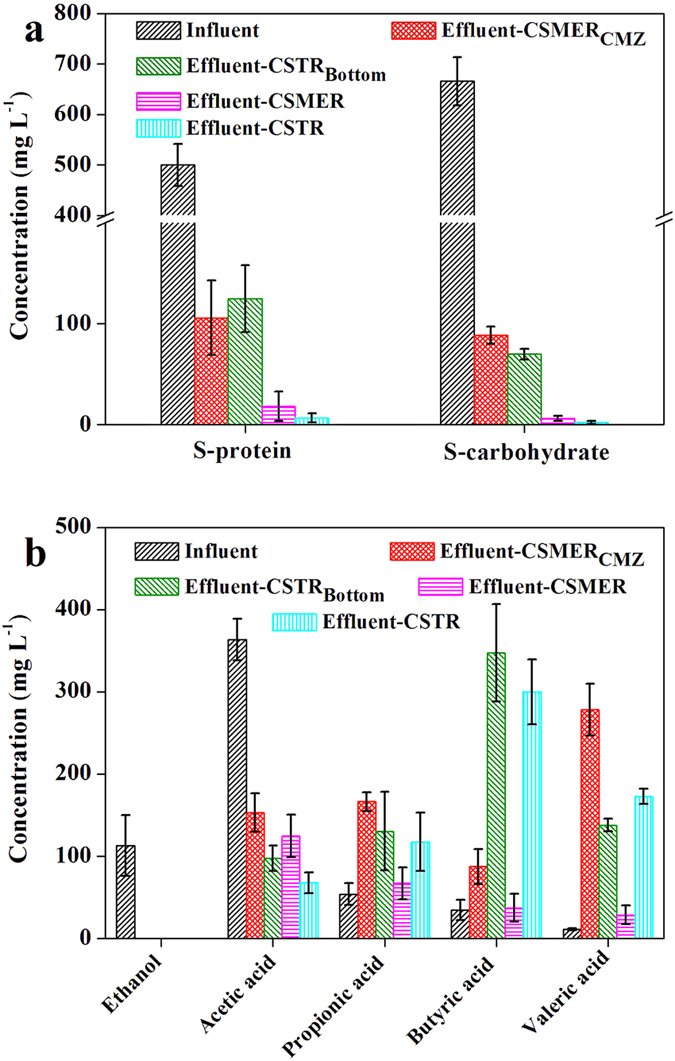
Changes in (**a**) s-protein and s-carbohydrate and (**b**) VFAs during cascade degradation in CSMER and CSTR in Phase III.

Brewery wastewater is usually comprised of various soluble organic compounds in the form of soluble proteins (s-proteins), soluble carbohydrates (s-carbohydrates) and volatile fatty acids (VFAs). In order to determine the process by which substrates were degraded in CSMER, the composition and concentration of soluble organic matter were detected in brewery wastewater. The CSMER removed 96.3 ± 3.2% of s-protein and 99.1 ± 1.8% of s-carbohydrates. Also, CMZ degraded 79.1 ± 5.6% of s-protein and 86.6 ± 2.2% of s-carbohydrates by anaerobic digestion process with short-chain VFAs as main products, which were regarded easier for exoelectrogens to generate electricity in the MEZ (Fig. 4a). The total VFAs concentration first increased to 694 ± 82 mg L^−1^ in the CMZ effluent then decreased to 257 ± 35 mg L^−1^ in the final effluent.

Ethanol, acetic acid, propionic acid, butyric acid and valeric acid were the main VFAs in raw brewery wastewater. Ethanol was exhausted after the anaerobic digestion in CMZ. The acetic acid kept declining while other VFAs first accumulated in the CMZ and then decreased in the MEZ. Valeric acid accumulated in the largest scope, with an increase in the concentration from 10 ± 1 mg L^−1^ to 278 ± 31 mg L^−1^. Accumulation of propionic and butyric acid were also observed, whose concentrations increased from 53 ± 13 mg L^−1^ to 166 ± 11 mg L^−1^ and 34 ± 12 mg L^−1^ to 87 ± 21 mg L^−1^, respectively (Fig. 4b). These accumulated VFAs and remaining complex organics (s-protein and s-carbohydrates, 105 ± 36 and 88 ± 9 mg L^−1^, respectively) were then utilized in the MEZ for electricity generation, thereby, not only recovering energy but also polishing effluent quality. During the parallel operation of CSTR, though nearly the same s-protein and s-carbohydrates removal efficiencies (98.7 ± 3.8% and 99.6 ± 1.2%, respectively) were achieved, total VFAs concentration in the CSTR effluent was 2.5 times higher than that of CSMER. Concentration of propionic acid, butyric acid and valeric acid significantly increased in the CSTR effluent and few VFAs were removed neither in the bottom nor upper part of CSTR, revealing that degradation in the CSTR was not complete. Detection of hydrogen (0.18 ± 0.04 L L^−1^ d^−1^) in the CSTR showed that its fermentation pathway was favorable for hydrogen production, consequently, leaving the remainder of organic matter present as VFAs.

### Spatial distribution of microbial community

#### Bacterial community

Pyrosequencing was used to characterize microbial communities in CSMER and CSTR. The five samples taken from CSMER (CSMER_CMZ_, CSMER_Anode_ and CSMER_cathode_) and CSTR (CSTR_Bottom_ and CSTR_Up_) yielded qualified sequencing reads in the range of 15453 to 33094, with each sample clustered to more than one thousand operational taxonomic units (OTUs) based on a threshold of 97% (Table S2). CSMER_Cathode_ had the highest diversity (Shannon = 5.95), and was slightly larger than that of CSTR_Bottom_ and CSTR_Up_ (Shannon = 5.89, 5.69), while CSMER_Anode_ had the lowest diversity (Shannon = 4.88). Rarefaction curves showed that new phylotypes would continue to merge even after 20,000 reads, as none of the curves tended to reach a plateau (Figure S2). Coverage value of each sample was more than 0.95, suggesting adequate sampling for the assessment of community composition^26^.

**Figure 5:**
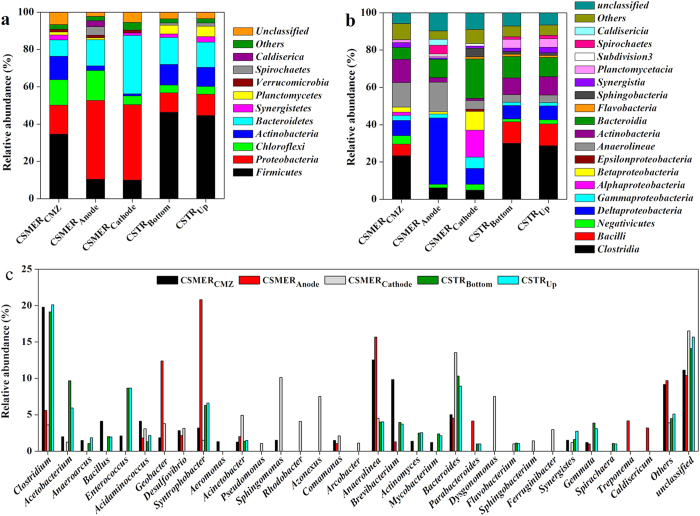
Relative abundance of bacterial reads retrieved from the CSMER_CMZ_, CSMER_Anode_ , CSMER_Cathode_, CSTR_Bottom_ and CSTR_Up_ classified at the (**a**) phylum (**b**) class and (**c**) genus level. Others refer to the phylum, class and genus with a relative abundance less than 1%.

Qualified reads retrieved from CSMER_CMZ_, CSMER_Anode_, CSMER_cathode_, CSTR_Bottom_ and CSTR_Up_ were assigned to known phyla, classes and genera (Fig. 5). The five samples mainly belonged to ten phyla, and the majority of phyla were *Firmicutes* (9.9–46.4%)*, Proteobacteria* (10.3–42.3%) and *Bacteroidetes* (8.8–31.3%) (Fig. 5a). At the class level, the CSMER_CMZ_ bacterial community was dominated by classes *Clostridia* (23.3%), *Anaerolineae* (13.1%), *Actinobacteria* (12.5%) , *Deltaproteobacteria* (8.1%) and *Bacteroidia* (6.4%). The CSMER_Anode_ community composition was different from that of CSMER_CMZ_, which was dominated by *Deltaproteobacteria* (35.5%), followed by *Anaerolineae* (15.9%), *Bacteroidia* (9.8%) and *Clostridia* (6.2%). Since most exoelectrogens such as *Geobacter* belong to *Deltaproteobacteria*, bacteria attached to anode might be mainly for electricity generation^27^. The cathode of the CSMER mostly reflected *Bacteroidia* (21.3%), with less amounts of *Alphaproteobacteria* (14.5%), *Betaproteobacteria* (10.2%) and *Deltaproteobacteria* (8.5%). The CSTR_Bottom_ and CSTR_Up_ were possessed of an approximately same structure at the class level with the predominance of *Clostridia* (30.1%, 28.7%), *Bacilli* (11.4%, 11.8%), *Bacteroidia* (11.5%, 10.3%) and *Actinobacteria* (9.1%, 9.9%) (Fig. 5b).

Genus level identification allowed us to further examine the reactors performance based on bacterial function (Fig. 5c). The most frequently identified sequences in CSMER_CMZ_ were assigned to *Clostridium* (19.7%), *Anaerolinea* (12.5%), *Brevibacterium* (9.9%) and *Bacteroides* (5.0%). Similarly, *Clostridium* (19.1%) and *Bacteroides* (10.3%) were also the two major genera in CSTR_Bottom_. Certain species of the genus *Clostridium*, such as *Clostridium cellobioparum* and *Clostridium sufflavum* are highly involved in polysaccharides degradation both in laboratory and full-scale anaerobic reactors treating various wastewaters^28^,^29^. Moreover, *Clostridium* spp. is a predominant hydrolytic bacterium on the surface layer of brewery-degrading granule^30^. The *Bacteroides* genus is an important mesophilic fermentative bacterium that play a role in sugar catabolism, with major products of hydrogen, carbon dioxide and lower fatty acids^16^. Apart from *Clostridium* and *Bacteroides*, diverse fermentative bacteria including *Bacillus*, *Acetobacterium* and *Enterococcus* also existed in CSMER_CMZ_ and CSTR_Bottom_^31^,^32^. These observations suggested that bacterial communities in CSMER_CMZ_ and CSTR_Bottom_ played a key role in the primary hydrolysis and acidification of macromolecular organic compounds, revealing by the fact that most s-protein and s-carbohydrates in brewery wastewater were degraded in the CMZ of CSMER and the bottom zone of CSTR. The CSMER_Anode_ community was most similar to *Syntrophobacter* (20.8%), *Anaerolinea* (15.6%) and *Geobacter* (12.4%), which was much different from that of CSTR_Up_*. Geobacter*, which was the most predominant known exoelectrogen, was found present only in CSMER, suggesting that exoelectrogens have a competitive advantage over other bacteria when current was generated^33^. The *Syntrophobacter* genus is a syntrophic bacteria capable of oxidizing VFAs to formate, acetate and hydrogen, which is always in coculture with hydrogen or formate-utilizing methanogens^34^. The existence of *Geobacter* would be one reason for more *Syntrophobacter* presenting in CSMER_Anode_, since certain species of *Geobacter* was hydrogen consuming organism^35^,^36^. The relatively more abundant *Syntrophobacter* present in CSMER_Anode_ (20.8%) compared with CSTR_Up_ (6.6%) meant that more VFAs could be removed by CSMER, consequently, polishing the effluent quality. Some well-known ammonia-oxidizing bacteria, such as *Nitrosomonas* and *Nitrospira*^37^ were not detected in CSMER_Cathode_, consequently, nitrification was rarely detected in the CSMER. This finding was not in accordance with results from previous studies that found the microorganisms on the cathode of a sediment MES participated in ammonia oxidation^38^. Alterations of oxygen level near the cathode surface caused by cathodic oxygen reduction might have impact on microbial community structure.

#### Archaeal community

**Figure 6:**
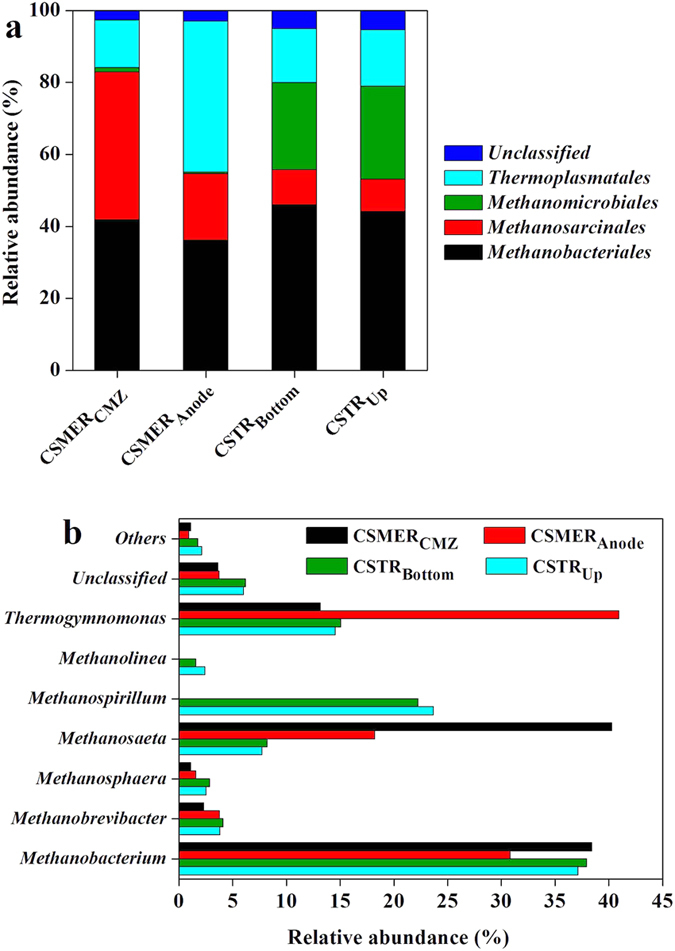
Relative abundance of archaeal reads retrieved from the CSMER_CMZ_, CSMER_Anode_ , CSTR_Bottom_ and CSTR_Up_ classified at the (**a**) order and (**c**) genus level. Others refer to the order and genus with a relative abundance less than 1%.

Archaeal community analysis based on alpha diversity resulted in 1388 (CSMER_CMZ_), 895 (CSMER_Anode_) OTUs for the CSMER, and 1491 (CSTR_Bottom_), 1342 (CSTR_Up_) OTUs for the CSTR (Table S3). Rarefaction curves based on a 97% similarity also did not reach a plateau (Figure S3). The order level identification of the archaeal community showed that both hydrogenotrophic (*Methanobacteriales*, 41.8%) and acetoclastic methanogens (*Methanosarcinales*, 41.1%) were abundant in CMZ of CSMER (Fig. 6a). However, the CSTR_Bottom_ and CSTR_Up_ were dominated by hydrogenotrophic methanogens (*Methanobacteriales* and *Methanomicrobiales*). The anode of CSMER mostly reflected *Thermoplasmatales* (41.9%), with less amounts of *Methanobacteriales* (36.2%).

At the genus level, the predominant genera in CSMER_CMZ_ were affiliated with *Methanosaeta* (40.3%), *Methanobacterium* (38.4%) and *Thermogymnomonas* (13.2%) (Fig. 6b). In the CSTR, *Methanosaeta* was much less abundant in CSTR_Bottom_ and CSTR_Up_ (8.2% and 7.8%), with the main archaeal genera belonging to *Methanobacterium* (37.9% and 37.1%) and *Methanospirillum* (22.3% and 23.7%). Since *Methanosaeta* have been found to dominate under stable reactor with high biogas production rate and methane yield, the high relative abundance of *Methanosaeta* in CSMER_CMZ_ indicated a favorable operation condition for methane production^39^. These findings were consistent with the higher methane production rate in CSMER compared with CSTR, which might be caused by synergistic effects between microbial communities between CMZ and MEZ. *Methanobacterium* (30.8%) and *Methanosaeta* (18.2%) were also observed on anode of CSMER. The co-existence of methanogens and exoelectrogens on anode of MEZ adversely affected electricity production, since methanogenesis diverted energy away from electrogenesis, consequently, reducing CE of the CSMER^22^.

The microorganisms in CSMER could be functionally categorized into four groups, including fermentative bacteria (*Clostridium, Bacteroides*), acetogenic bacteria (*Syntrophobacter*), methanogenic archaea (*Methanosaeta* and *Methanobacterium*) and exoelectrogens (*Geobacter*), which distributed spatially in CMZ and MEZ of the system. The clear spatial distribution and complex syntrophic interactions of these four groups drove the CSMER to cascade degrade a wider range of substrates and exhibit better wastewater treatment efficiency compared with a control CSTR. Firstly, pre-hydrolyzing and fermenting of macromolecular organic compounds by fermentative bacteria in the CMZ were responsible for high power generation obtained in CSMER using brewery wastewater. Effective conversion of the s-proteins and s-carbohydrates contained in brewery wastewater to hydrogen, carbon dioxide and short-chain fatty acids by *Clostridium*, *Bacteroides* and other diverse fermentative bacteria in the CMZ was the first step for cascade degradation process. Products of the fermentation stage, such as short-chain fatty acids and amino acids were oxidized to acetate, formate, hydrogen and carbon dioxide by *Syntrophobacter*, which were better-utilizable substrates that could be further utilized by *Methanosaeta* and *Methanobacterium* for methane production, or transferred to MEZ for electricity generation by *Geobacter*. Secondly, electricity generation by *Geobacter* in MEZ made the CMZ a more suitable niche for methane production. A large portion of the VFAs produced in the CMZ were transferred to MEZ along the hydraulic path and utilized by *Syntrophobacter* and *Geobacter* on the anode of CSMER, consequently, releasing their inhibition on methanogens. Since the removal of VFAs from anaerobic digestion of organic matter could accelerate their eventual decomposition to CO_2_ and H_2_O^40^,^41^, the relatively higher *Syntrophobacter* and *Geobacter* in MEZ could contribute to higher methane production rate in CSMER. Actually, real wastewater such as brewery and winery wastewater cannot be efficiently used by exoelectrogens because of a high fraction of particulate and fermentative substrate^14^. Therefore, the co-existence of various functional microbial communities as well as a clear spatial distribution and syntrophic interaction among them were crucial for MES-centered systems towards practical application.

#### Significance of the CSMER for wastewater treatment

There have been growing interests in combining AD and MESs for wastewater treatment, due to the complementary synergy between these two processes. However, some studies just connected two individual reactors in sequence without truly integrating them while the CSMER was a truly integrated system by integrating continuous stirred tank reactor (CSTR) and microbial electrochemical system into a single design. TCOD loading rate in the CSMER was 7.4 kg COD m^−3^ d^−1^, which was 2.5 times higher than that of an upflow anaerobic sludge blanket (one of the most effective anaerobic processes for brewery wastewater treatment)^4^. Moreover, TCOD degradation rate in CSMER was 10 times higher than that in a 90-liter stackable baffled MES (0.5 kg COD m^−3^ d^−1^)^12^. Though the mean electricity output obtained in the 90-liter stackable baffled MES (60 mA) was much higher than that of CSMER (12 mA), energy recovery in the CSMER (0.682 kWh m^−3^) was seven times higher of the 90-liter stackable baffled MES (0.097 kWh m^−3^) because of additional gaseous methane energy recovered by CSMER (detailed calculations in the supporting information). This fact is important for the application of MES-centered hybrid system in wastewater treatment, because technologies characterized by energy efficient and energy recovery are beneficial for environmental sustainability.

Though several MES-centered hybrid systems have been previously reported, the CSMER held distinct advantages on the basis of capital costs, wastewater treatment and electricity generation (Table 1). Firstly, poor economic viability was preliminarily resolved in CSMER due to its membrane-less design compared with SMFC, UMFC and AFB-MFC^42^,^43^,^44^. Capital cost was further reduced by using rolling-pressed activated carbon cathode, which was much cheaper than platinum-coated carbon cloth cathode^45^,^46^. Furthermore, attributed to the addition of anaerobic activated sludge, the CSMER was more suitable for treating suspended organic matters compared with some AD-MESs systems using felts, granules and meshes of carbon as anodic electrode materials^47^. Thirdly, high rate of pre-hydrolysis and acidification can be achieved in the CSMER due to continuous stirring effects and solid-liquid-gas separation, which were considered to be the rate limiting step for bioelectricity generation.

Though remarkable advantages of the CSMER were shown here, additional work will be needed towards its practical implementation of wastewater treatment. Effluent TCOD (909 ± 296 mg L^−1^) was unfavorable for aerobic post-treatment step because of high concentration, therefore, performance in terms of substrate removal need to be optimized by adjusting HRT, reducing external resistance, regulating functional microbial communities or adding pre-treatment step. In addition, assessment of nitrogen and phosphorus removals was supposed to be conducted to evaluate its nutrient removal efficiency. Moreover, future research focusing on enhancing energy recovery efficiency (such as electrode modification) would benefit MES technology for practical implementation of wastewater treatment.

In the present study, a CSMER showed great potential for practical implementation when treating brewery wastewater, which achieved 75.4 ± 5.7% of TCOD removal and 64.9 ± 4.9% of TSS removal. Cascade utilization of organic matter by CMZ and MEZ in CSMER contributed to its higher substrate removal efficiency compared with a control CSTR. Pyrosequencing analysis demonstrated that four groups of microorganisms, including fermentative bacteria, syntrophic acetogenic bacteria, methanogenic archaea and electrochemically active bacteria participated in this cascade degradation process. This hybrid system shows a high economical attractiveness and practical applicability due to its membrane-less design and use of cost-effective materials.

## Methods

### Brewery wastewater

Brewery wastewater was obtained from a local beer brewery in Harbin (Heilongjiang, China). The raw wastewater contained total chemical oxygen demand (TCOD) of 3707 ± 220 mg L^−1^, soluble chemical oxygen demand (SCOD) of 2466 ± 189 mg L^−1^, biochemical oxygen demand (BOD) of 2064 ± 118 mg L^−1^, total suspended solids (TSS) of 1546 ± 136 mg L^−1^ and volatile suspended solids (VSS) of 579 ± 53 mg L^−1^. The content of soluble carbohydrates (s-carbohydrates), soluble proteins (s-proteins) and volatile fatty acids (VFAs) were 665 ± 47, 500 ± 41and 575 ± 131 mg L^−1^ respectively. BOD/COD value was about 0.58 indicating that the wastewater was easily biodegradable. Conductivity on the other hand was 3.2 mS cm^−1^, making it suitable for microbial electrochemical systems for electricity generation.

### CSMER construction and operation

**Figure 7:**
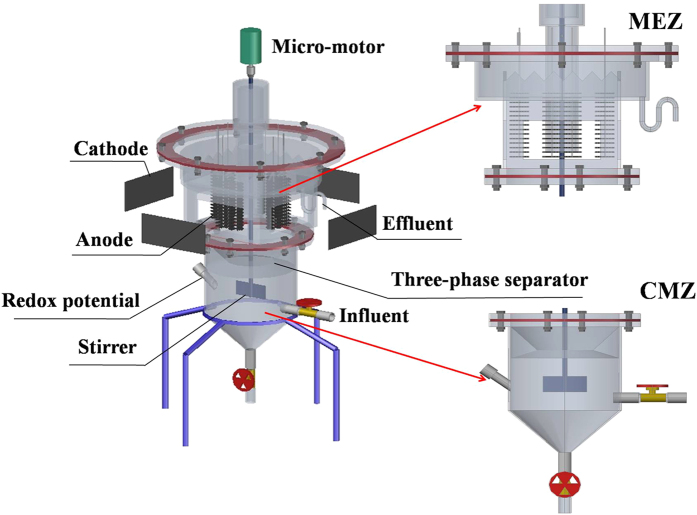
Schematic diagram of the CSMER (CSMER: continuous stirred microbial electrochemical reactor, CMZ: complete mixing zone, MEZ: microbial electrochemical zone).

The CSMER was constructed from plexiglas and comprised of a complete mixing zone (CMZ) and a microbial electrochemical zone (MEZ), with the total working volume of 4 L^17^ (Fig. 7) (Figure S4). The CMZ was a cylindrical chamber (ID 175 mm × H 94 mm) locating at the bottom of CSMER, and the rectangular MEZ (H 125 mm × 175 mm × 175 mm) was positioned on the top of the reactor. The two functional zones were separated by a three-phase separator, which was concomitantly used for solid-liquid-gas separation and gas collection. Continuous mixing of the CMZ was achieved by a micro-motor installation on the upper portion of the MEZ. Twelve carbon fiber brushes (4 cm in diameter and 10 cm in length, Toray, 3 K carbon fiber) pretreated at 450 °C for 30 min as previously reported^48^ were fixed vertically in a square on the upper portion of the MEZ, with every three forming a row and separating by four porous plexiglas plates. Four rolling-pressed activated carbon cathodes^49^ (each has a projected surface area of 58 cm^2^) were fixed on each face of the quadrangle MEZ. They were supported with a plexiglas plate, which was perforated to facilitate oxygen transfer. The MEZ was divided into four independent cells, each comprising of a three-carbon fiber brush-anode and a piece of rolling-pressed activated carbon cathode.

The CSMER was inoculated with 1 L of anaerobic activated sludge collected from a continuous stirred-tank reactor treating cellulosic ethanol wastewater and had been operated for 120 days with sucrose medium as feed. The system was operated in continuous-flow mode with each circuit connected to a 10 Ω external resistor during this experiment, which was the optimized parameters according to the previous research^17^. In order to keep a constant influent loading rate and avoid sudden increased inhibitor in real wastewater, tests were conducted in three phase by increasing brewery wastewater concentration gradients (30%, 60% and 100% in volume in each phase, V/V) in the feed, at the same time, sucrose was added to maintain the influent COD around 3707 ± 220 mg L^−1^ (2.25, 1.28 and 0 g L^−1^ in each phase) and 50 mmol L^−1^ PBS was added to maintain the influent conductivity around 3.2 mS cm^−1^ (0.32, 0.18 and 0 L L^−1^ in each phase). The system operated in each phase for 20 days to ensure steady performance with the whole experiment lasting for nearly 60 days. During Phase III, power production and wastewater treatment efficiencies were assessed by measurement of maximum power density, COD removal, SS removal and biogas production rate. Concentration of s-carbohydrates, s-proteins and VFAs in both zones were also monitored during Phase III to evaluate cascade utilization of substrates in this system. A continuous stirred tank reactor (CSTR) operated in parallel as a control.

### Measurements and calculations

The cell voltage was recorded using PISO-813 data acquisition system (32 Channel ICP DAS Co., Ltd.) every 30 min. Polarization curves and coulombic efficiency (CE) were obtained as described previously^50^. The electrochemical analyses were conducted at the end of each phase with a workstation (Autolab PGSTAT128N, Metrohm Co., Swiss). Cyclic voltammetry (CV) was performed with the anode as working electrode, cathode as counter electrode and a saturated calomel electrode (SCE, +0.214 V vs. SHE) as reference electrode at a scan rate of 1 mV s^−1^ in the potential range of −0.8 V to + 0.2 V. Electrochemical impedance spectroscopy (EIS) were carried out with cathode, anode and SCE as the working electrode, counter electrode and reference electrode over a frequency range of 100 kHz to 10 mHz with the amplitude of 10 mV. Prior to each measurement, the system operated under open circuit condition for more than 12 h until the open circuit voltage was stable and the CV was conducted under non-turnover condition^51^.

TCOD, SCOD, TSS and VSS were measured according to Standard Methods (APHA 1998). Both VFAs and biogas composition were analyzed by a gas chromatograph (Agilent GC 7890A, USA)^52^. S-proteins were measured using a Bicinchoninic Acid Assay (BCA Protein Assay Kit, Sangon Biotech) according to the instructions. S-carbohydrates were determined by phenol-sulfuric method^53^. All samples used for SCOD, VFAs, s-proteins and s-carbohydrate analysis were filtered through a 0.45 μm pore diameter syringe filter.

### Microbial community analysis

The microbial communities of CSMER_CMZ_, CSMER_Anode_, CSMER_cathode_, CSTR_Bottom_ and CSTR_Up_ were analyzed by pyrosequencing. Total genomic DNA was extracted from all samples using a Bacteriag DNA Mini Kit (Watson Biotechnologies, Inc., Shanghai) according to the manufacturer’s instructions and assessed by electrophoresis in 1% agarose gels. The bacterial 16S rDNA PCR and sequencing was performed using 341F and 805R Primers targeting the variable region V1–V3. Archaeal 16S rDNA Nested PCR and sequencing were performed using two pairs of PCR primers, which were 340F-1000R and 349F- 806R. Pyrosequencing of amplicons was performed by Sangon Biotech Company using 454/Roche GS-FLX instrument as previously described^16^.

## Additional Information

**How to cite this article**: Wang, H. *et al*. Cascade degradation of organic matters in brewery wastewater using a continuous stirred microbial electrochemical reactor and analysis of microbial communities. *Sci. Rep*. **6**, 27023; doi: 10.1038/srep27023 (2016).

## Supplementary Material

Supplementary Information

## Figures and Tables

**Table 1:** Performance and characteristics of systems integrating MES and conventional anaerobic treatment process.

Integrated system	Anaerobic reactor (AD)	Upflow Anaerobic Sludge Blanket (UASB)	Anaerobic fluidized bed (AFB)	Anaerobic baffled reactor (ABR)	Anaerobic fluidized bed membrane bioreactor (AFMBR)	Continuous stirred tank reactor (CSTR)
Wastewater	Sewage sludge	Sucrose wastewater	Diluted alcohol distillery wastewater	Diluted liquid from corn stover steam explosion process	Domestic wastewater	Brewery wastewater
Influent COD (mg L^−1^)	49700 ± 2700	1000	10476.19	7160 ± 50	210 ± 11	3707 ± 220
COD removal	78.1%	90%	80-90%	89.1%	92.5%	75.4%
Maximum power density	145 ± 5 mW m^−2^	170 mW m^−2^	124.03 mW m^−2^	10.7 W m^−3^	~89 mW m^−2^ for each MFC	304 ± 32 mW m^−2^ for each cell
Anode	Carbon paper	Reticulated vitreous carbon	Carbon fiber paper	Graphite plate and graphite granules	Graphite fiber brushes	Carbon fiber brushes
Cathode	Platinum-coated carbon paper	Reticulated vitreous carbon	Carbon fiber paper	Platinum-coated carbon cloth	Platinum-coated carbon cloth	Rolling-pressed activated carbon cathodes
Membrane	Nafion 117 PEM	PEM	Nafion 117 PEM	No	No	No
Working volume	0.6 L	0.77 L	9.19 L	0.42 L	0.13 L for each MFC reactor and 65 mL for the AFMBR	4.00 L
Characteristic	Capable of treating raw sludge	Achieve higher than 90% of SCOD removal and leave the effluent VFA lower than 100 mg L^−1^	Capable of treating high strength wastewater at an OLR of 16.86 Kg COD m^−3^ d^−1^; Use carriers for biofilm growth	The volumetric loading rate is as high as 29.5 KgCOD m^−3^ NAC d^−1^	Produce a high effluent quality with a near neutral energy requirement	Truly integration system; Use anaerobic activated sludge
Reference	[44]	[42]	[43]	[45]	[46]	This work
