# Supplementary material for: Cascade degradation of organic matters in brewery wastewater using a continuous stirred microbial electrochemical reactor and analysis of microbial communities
Source: Sci Rep. 2016 Jun 7;6:27023. doi: 10.1038/srep27023 (PMC4895234; doi:10.1038/srep27023)
Supplement: Supplementary Information [file srep27023-s1.doc]

**Appendix A. Supplementary data**

**Cascade degradation of organic matters in brewery wastewater using a continuous stirred microbial electrochemical reactor and analysis of microbial communities**

Haiman Wang^a^, Youpeng Qu^a,b**^, Da Li^a^, John J. Ambuchi^a^, Xiangtong Zhou^a^,

Jia Liu^a^ & Yujie Feng^a*^

^a^State Key Laboratory of Urban Water Resource and Environment, Harbin Institute of Technology. No. 73 Huanghe Road, Nangang District, Harbin 150090, China

^b^School of Life Science and Technology, Harbin Institute of Technology. No. 2 Yikuang Street, Nangang District, Harbin 150080, China

*Corresponding Author:

E-mail: [yujief@hit.edu.cn](mailto:yujief@hit.edu.cn); phone: (+86)451-86287017;

Fax: (+86) 451-86287017

** Co-Corresponding Author:

E-mail: [pp198259@hit.edu.cn](mailto:pp198259@hit.edu.cn)

Table S1 Performance of CSMER and CSTR during three phase operation

|  | Current  (m A) | | | | PD_max_^a^  (m W m^–2^) | | | | CE^b^  (%) | TCOD removal_CSMER_  (%) | SCOD removal_CSMER_  (%) | TCOD removal_CSTR_  (%) | SCOD removal_CSTR_  (%) |
| --- | --- | --- | --- | --- | --- | --- | --- | --- | --- | --- | --- | --- | --- |
|  | C1 | C2 | C3 | C4 | C1 | C2 | C3 | C4 |  |  |  |  |  |
| Phase I | 17.8±1.5 | 17.6±1.4 | 16.4±1.9 | 18.1±1.6 | 536 ± 9 | 519±18 | 512±23 | 541±22 | 1.9±0.3 | 83.2 ± 1.3 | 80.0 ± 3.1 | 53.8 ± 6.7 | 43.9 ± 8.9 |
| Phase II | 14.7±0.7 | 14.3±1.7 | 13.7±1.9 | 14.4±1.2 | 457± 8 | 429±36 | 412±12 | 464±14 | 1.6±0.4 | 79.1± 3.4 | 76.8 ± 3.5 | 49.6 ± 3.4 | 41.6 ± 3.8 |
| PhaseIII | 12.8±2.0 | 11.4±1.5 | 10.6±1.4 | 13.2±1.2 | 313±32 | 289±34 | 273±14 | 344±31 | 1.5±0.5 | 75.4 ± 5.7 | 73.1 ± 4.8 | 47.2 ± 6.9 | 38.4 ± 5.1 |

^a^ Maximum power density

^b^ CE was calculated based on TCOD which was consumed in the whole CSMER


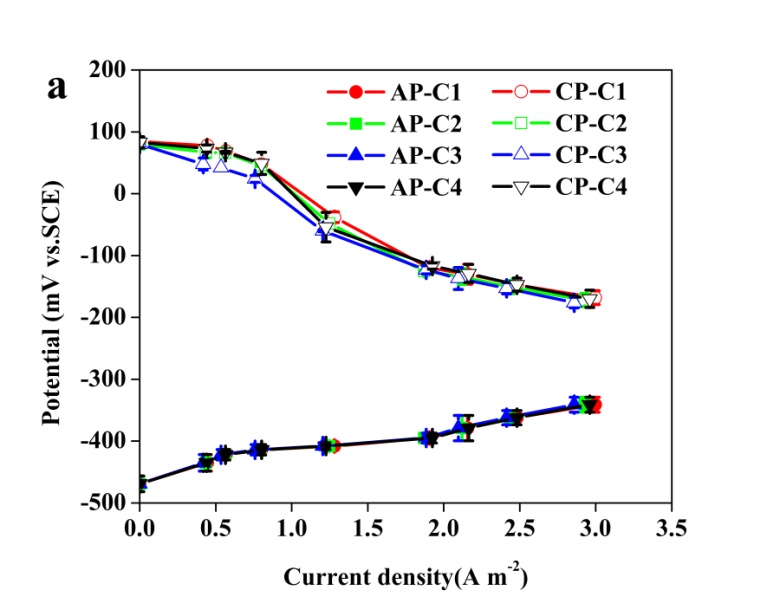

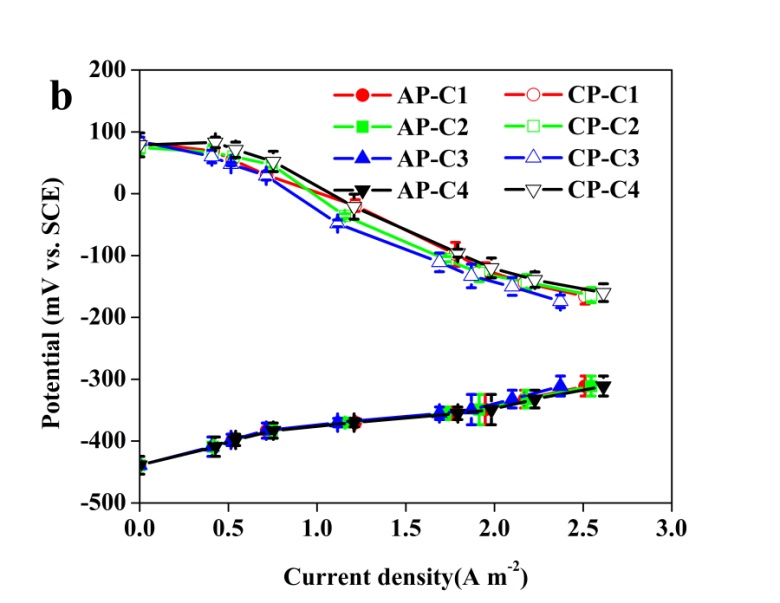


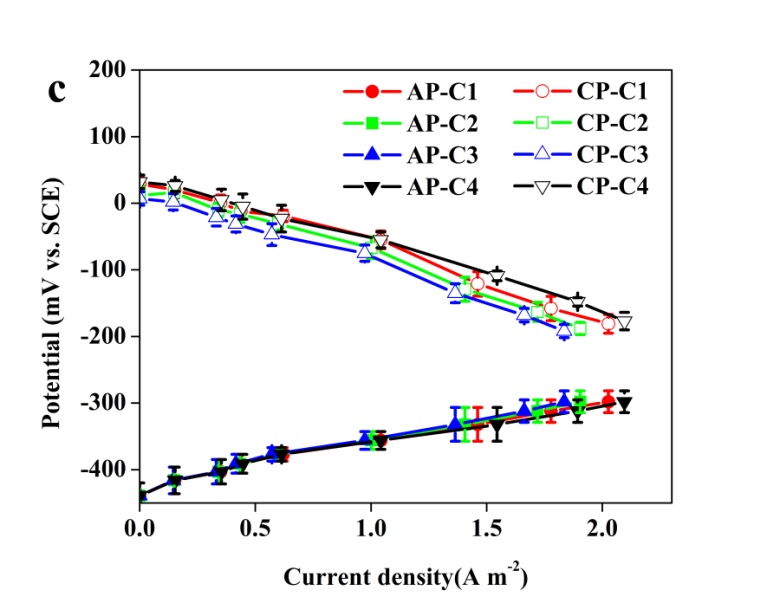


Figure S1. Electrode potentials as a function of current density for CSMER in (a) Phase I (b) Phase II and (c) Phase III (AP: anode potential, CP: cathode potential, C1: Cell 1, C2: Cell 2, C3: Cell 3, C4: Cell 4)

Table S2 Estimators for evaluation of bacterial community diversity and richness

| Samples | Reads | OTU | ACE | Chao 1 | Shannon | Coverage |
| --- | --- | --- | --- | --- | --- | --- |
| CSMER_CMZ_ | 15453 | 1216 | 2956.183 | 2203.242 | 4.96668 | 0.96182 |
| CSMER_Anode_ | 19417 | 1519 | 5086.250 | 3406.781 | 4.88306 | 0.956584 |
| CSMER_Cathode_ | 20134 | 1735 | 3520.809 | 3010.698 | 5.954901 | 0.962849 |
| CSTR_Bottom_ | 33094 | 3150 | 11308.951 | 7190.761 | 5.894497 | 0.954914 |
| CSTR_Up_ | 31028 | 2876 | 10091.257 | 6889.562 | 5.689524 | 0.955728 |

Table S3 Estimators for evaluation of archaeal community diversity and richness

| Samples | Reads | OTU | ACE | Chao 1 | Shannon | Coverage |
| --- | --- | --- | --- | --- | --- | --- |
| CSMER_CMZ_ | 20173 | 1388 | 3664.008 | 2666.621 | 4.036045 | 0.964259 |
| CSMER_Anode_ | 17387 | 895 | 2340.840 | 1741.138 | 3.417909 | 0.972278 |
| CSTR_Bottom_ | 43138 | 1491 | 3390.295 | 2738.75 | 3.687729 | 0.983448 |
| CSTR_Up_ | 41216 | 1342 | 3114.465 | 2613.97 | 3.615423 | 0.972357 |


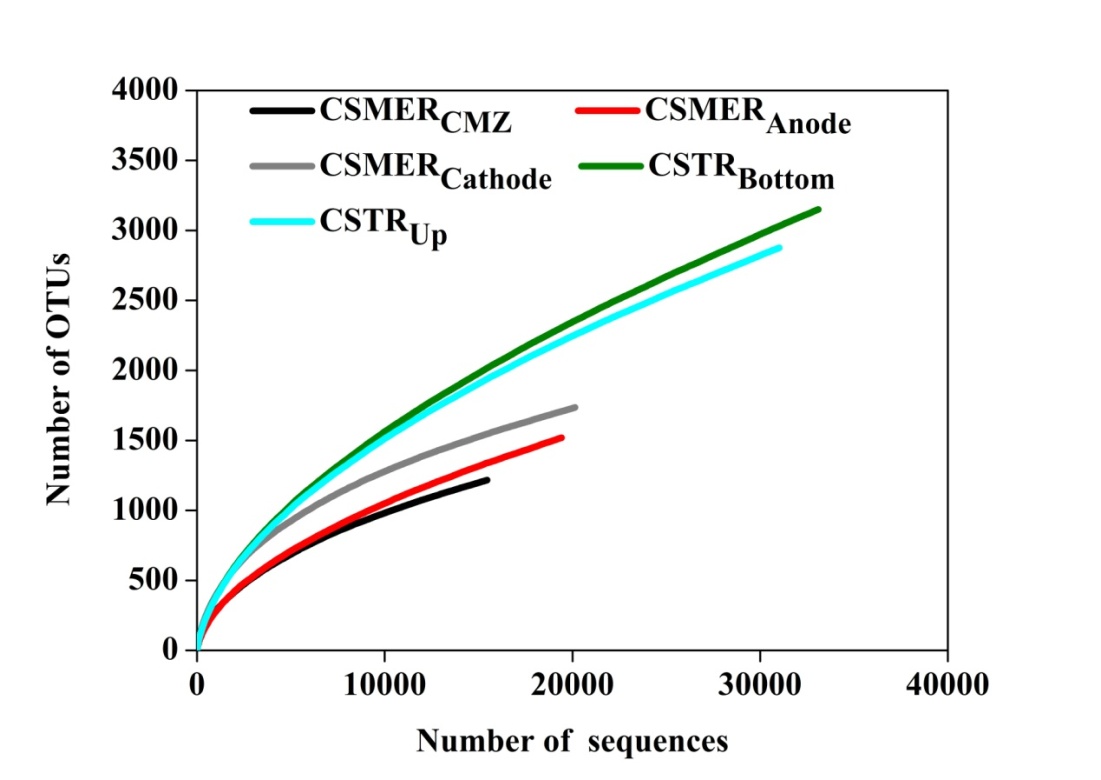


Figure S2 Rarefaction curves based on pyrosequencing of the bacterial communities


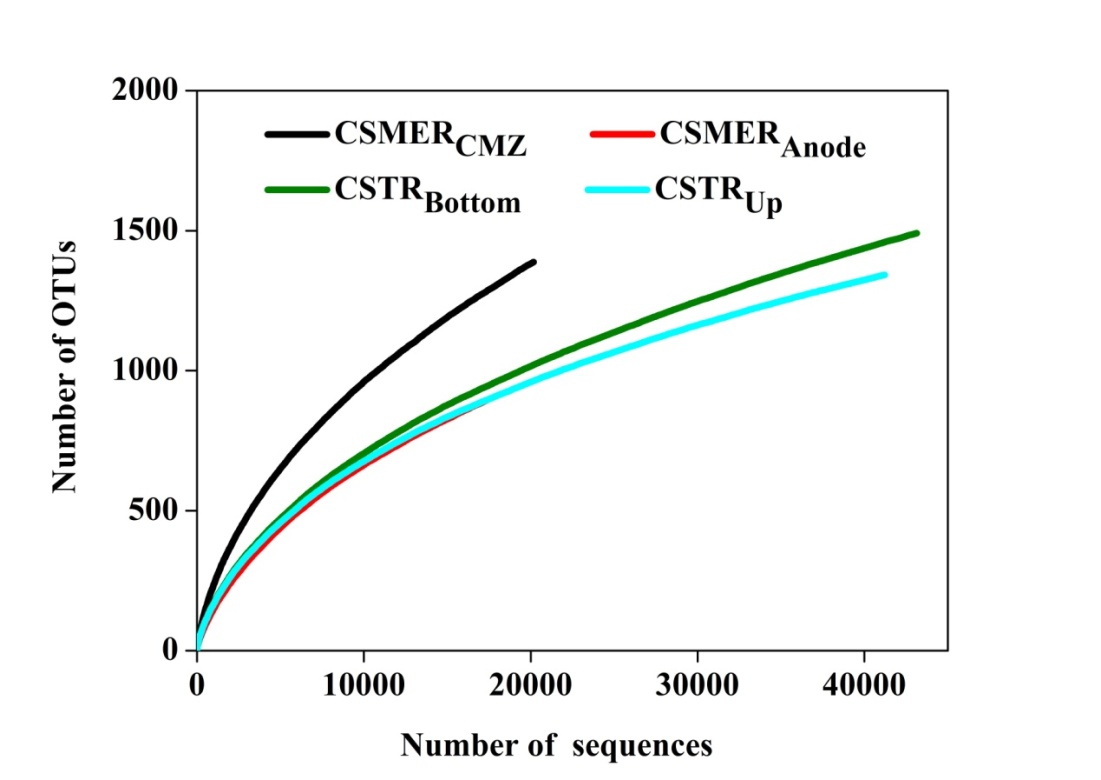


Figure S3 Rarefaction curves based on pyrosequencing of the archaeal communities


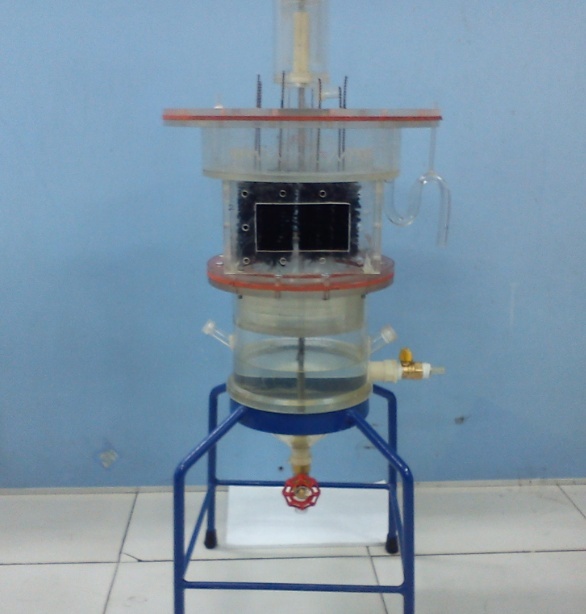


Figure S4 Photo of the continuous stirred microbial electrochemical reactor

**Energy recovery of the CSMER**

Energy recovery (ER) was expressed as kilowatt hour per cubic meter of influent wastewater (kWh m^–3^) according to formula (1), (2) and (3).

$ER=ER_{\text{electric}}+ER_{\text{methane}}$ **(1)**

$ER_{\text{electric}}=\frac{4I^{2}Rt}{1000V_{\text{w}}}$ **(2)**

$ER_{\text{methane}}=\frac{Y_{\text{methane}}q_{\text{methane}}t\eta}{24\times3600V_{\text{w}}}$ **(3)**

I: mean current of each cell,1.2 × 10^–3^ A; R: external resistor of each cell, 10 Ω; *V*_w_: total working volume of the CSMER, 4 × 10^–3^ m^3^; *Y*_methane_: yield of methane, 1.62 ± 0.22 L d^–1^; *q*_methane_: heat of combustion of methane, 35.8 kJ L^–1^ CH_4_;η: efficiency of methane energy converted into electricity through combustion, 33 %
